# When Fathers Feel Socially Constrained to Assume a Role: A Negative Predictor of the Coparental Relationship in Switzerland

**DOI:** 10.3389/fpsyg.2021.752805

**Published:** 2022-01-03

**Authors:** Nicolas Favez, Aline Max, Michel Bader, Hervé Tissot

**Affiliations:** ^1^Faculty of Psychology and Educational Sciences, University of Geneva, Geneva, Switzerland; ^2^Department of Psychiatry, Lausanne University Hospital and University of Lausanne, Lausanne, Switzerland

**Keywords:** fathers, motivation, role distribution, cohesive coparenting, non-cohesive coparenting

## Abstract

Role distribution is a central issue for parents in the transition to parenthood, but little is known about the motivations in fathers to assume a specific role. Differences in work-family balance in each parent may be motivated by an individual choice mutually shared by both partners; however, in many couples, the parents may feel forced to adopt a traditional role distribution, either for financial reasons, or to comply with social expectations about what men and women should do when they are parents. This feeling of being socially constrained to adopt a role distribution that is not congruent with intrinsic motivations can generate dissatisfaction and may jeopardize the development of the interparental relationship. Coparenting refers to the emotional and instrumental support parents bring to each other in their parental tasks. It has been shown to be central in family functioning and a powerful predictor of children’s emotional and cognitive development. In this study, we aimed to assess the extent to which different motivations for role distribution in fathers are predictive of the quality of the coparental relationship. A convenience sample of 144 fathers from the French-speaking part of Switzerland completed online questionnaires about their motivations, coparental relationship, and sociodemographic characteristics. Results showed that the reasons for role distribution were mainly economical, practical, and in order to meet personal expectations. Multivariate general linear modeling showed that role distribution that is constrained to meet social expectations and age were predictive of a less cohesive coparental relationship, whereas a deliberate choice in role distribution was linked to a more cohesive coparental relationship.

## Introduction

Contemporary couples have to face important challenges, one of the most important of which is to reconcile family and professional life. This implies first, a sharing of the tasks between the two partners and second, for each partner to find a balance between family and work. These challenges are relatively new to couples: According to the so-called traditional family organization, roles are gendered, with the father taking the role of the breadwinner and the mother taking the role of the child caregiver and housekeeper, each parent assuming strictly separated tasks. This distribution follows the traditional model according to which women were naturally (i.e., biologically) determined to take care of children, and men were naturally determined to provide resources for the family through their work outside of the home (Cowan and Cowan, 1992; Maurer et al., 2001; Perälä-Littunen, 2007; Lamb and Lewis, 2010).

Following the gender revolution in the second half of the twentieth century, the possibility of interchangeability of tasks between mothers and fathers came to the front with the growth in women’s participation in the labor force. In the United States and in Western Europe, the increase in mothers’ work hours was paralleled by an increase in fathers’ participation in housework and childcare (National Institute of Child Health and Human Development (NICHD) Early Child Care Research Network, 2000; Gottfried et al., 2002; Jacobs and Kelley, 2006; Goldscheider et al., 2015; Frejka et al., 2018). However, the gender revolution is not accomplished yet: for example, the increase in the number of mothers in the workforce full time was not followed by a similar increase in the number of fathers being the primary caregiver (DeRose et al., 2019; Lee et al., 2020), and in dual-earner families, mothers still assume responsibility for most of the parenting duties, as social expectations regarding traditional roles are still present and influential (Yeung et al., 2001; Raley et al., 2012). This is particularly the case in Switzerland; in 2020, women still spent more time on domestic and family work than men did (28.7 vs. 19.1 h per week, respectively; Federal Statistical Office, 2021). Traditionally, Switzerland is a country where male employment is privileged and there is little direct help for families (compared with that in other Western European countries), so that the parental burden rests mainly on women (Jobin, 1995; Bonoli, 2007). As a consequence, parents may nowadays feel torn between the traditional roles they may be socially expected to endorse and the “new” roles that seem desirable to achieve equality. For fathers, combining involvement with children with their role as financial provider may be difficult, especially as the world of work still expects men to be fully dedicated to their work duties (McGill, 2014), and the lack of flexible work arrangements may constitute a structural barrier to increasing their involvement in the family (Carlson et al., 2021); on the other hand, men who become primary caregivers may have to face negative reaction from others who see them as “Mr. Mom” (Steinhour, 2018). Conversely, mothers engaged in the workforce have to face expectations of being a “good mother” dedicated to their children and simultaneously being competitive in professional work, this double agenda resulting in a burden that is heavier for women than it is for men (Craig, 2006; Borelli et al., 2017).

As a consequence, parents may have to face tensions within the family and between family and work. The latter work-family role conflict has been amply documented in the literature, role theory being one of the most influential explanations; that is, participation in one domain is made difficult by participation in the other (Goode, 1960). For example, a time-based conflict can occur when there are competing demands between the two domains; a strain-based conflict occurs when the stress met in one domain is carried over to the other, making the fulfillment of roles difficult in the second domain; and a behaviors-based conflict occurs when behaviors required by one domain make it difficult to fulfill the requirement of the other domain (Greenhaus and Beutell, 1985). Many individual and relational negative outcomes of work-family role conflicts have been described (Allen et al., 2000), such as marital distress, more negative and less positive communication styles between partners, parents’ depression, parent-child conflicts, and poorer physical and psychological health outcomes in children and parents (Bodenmann et al., 2007; Amstad et al., 2011; Hogan et al., 2014; Hill and Holmes, 2019). Several mechanisms explaining relational effects of work-family conflicts have been described, such as the spillover of work stress on both the parent-child relationship and the marital relationship (Perry-Jenkins et al., 2000; Bianchi and Milkie, 2010), or the crossover effect according to which the stress faced by one parent at work influences the relationship of the other parent with the child (Demerouti et al., 2005). However, roles are not necessarily conflictive; there may be a virtuous circle between family and work, as participation in a role may bring rewards (such as self-esteem) that may reinforce and enhance performance in the other role (Greenhaus and Powell, 2006).

Contextual factors (such as having a supportive job environment or a supportive family, the number of work hours, the degree of job security, the flexibility in the management of work hours, and the number of children and resulting burdens) have been shown to determine the extent to which an individual will have to face a work-family conflict or benefit from work-family enrichment (Michel et al., 2011b; Allen et al., 2013; Lapierre et al., 2018). However, more individual factors may also play a major role. Individual factors include the perception parents have of the workload or of stressors (a perception that is a strong moderator of the links between contextual factors and possible negative outcomes), the preference parents may have to work more or less hours than they actually do (Perry-Jenkins et al., 2000; Barnett and Hyde, 2001), and the extent to which task sharing and role endorsement meet the expectations of both parents (Fraenkel and Capstick, 2012). Indeed, whereas traditional sharing of tasks may meet their representations of parental roles (in some couples, both partners are intrinsically in favor of such an arrangement), in many couples, this arrangement is made for economic reasons or to comply with social expectations from the family of from the cultural environment; that is, it meets external factors but does not correspond to an intrinsic motivation. As a consequence, couple satisfaction may be affected negatively when partners feel constrained to adopt an unwanted role distribution (Amstad et al., 2011). Intrinsic motivation for parenting is one of the main predictors of paternal engagement (Lamb and Tamis-LeMonda, 2004). Whereas it has long been assumed that fathers are necessarily intrinsically motivated to be more invested in their work duties available data suggest that a significant portion of fathers at home have chosen to do so specifically to take care of their children and not only because they are unable to find a job (Lee et al., 2020). Moreover, some fathers may want to be engaged with their children and have expectations about parenthood (Goodman, 2005), but do not dare engage as much as they would like because of the negative reaction of their social environment (Steinhour, 2018). There are thus two complementary facets in paternal engagement: first, the motivation of fathers to be engaged in the family or to be mainly invested in their work, and, second, the fear they may have of the perceptions by others—their social environment and/or the mother—of the choice they have made (whatever this choice may have been). Little is known about the extent to which the renouncement by fathers of their primary and intrinsic motivation negatively affects the relationship with the mother, and, specifically, the coparenting duties.

Coparenting is indeed the specific part of the couple relationship that is dedicated to raising a child. It refers to the support parents bring to each other, at an instrumental and emotional level, in their parental duties (McHale, 2007). Cohesiveness is a central feature of the coparental relationship, that is, a relation that is marked by reciprocity, equity, mutual acknowledgment, and collaboration between the parents. A cohesive coparenting relationship is a favorable context for the emotional and cognitive development of the child (Teubert and Pinquart, 2010, for a meta-analysis). In a dissatisfying relationship, several configurations of non-cohesive coparenting may appear: the relation may be conflictive, with frequent unresolved disputes in which the child is the subject and with possible competition to gain the interest of the child; it may be skewed, one of the parents disengaging from coparenting and parenting duties; and it may be devitalized, as in a relation in which there is collaboration in everyday life but no emotional support or acknowledgment of the other parent’s efforts (Van Egeren and Hawkins, 2004; McHale and Lindahl, 2011). Coparenting consists of several dimensions in which cohesiveness may be implemented (Feinberg, 2003; Van Egeren and Hawkins, 2004; McHale, 2007; Feinberg et al., 2012): the child-rearing agreement, that is, the extent to which parents agree about parenting and education; support and undermining, that is, the emotional and instrumental support parents bring to each other or, on the contrary, the undermining of the other’s parenting through criticism and disparagement; the division of labor, that is, the effective sharing of tasks; the joint management of family dynamics, that is, the way parents manage relationships within the family; and finally, parenting-based closeness, that is, the sense of working as a team.

Relational processes are central in the occurrence and installation of non-cohesive patterns: dissatisfaction in the relationship is, for example, a strong predictor of coparental difficulties. A feeling of inequity or unfairness in task sharing or role distribution may thus alter the coparental relationship, as each parent may feel that she or he is giving more than the other (Milkie et al., 2002; Dew and Wilcox, 2011; DeMaris and Mahoney, 2017). Giving up the role for which one has a primary motivation and feeling constrained to endorse another role may be at the root of a possible feeling of inequity. The negative consequences of maternal dissatisfaction with task sharing and the feeling of inequity in the relationship with the father have been well documented (Goldberg and Perry-Jenkins, 2004; Moller et al., 2008); however, no study to date has examined the extent to which possible thwarted motivations in fathers may be linked with difficulties in the coparental relationship.

In the context of a larger study on parental burnout, we explored the motivations mentioned by fathers for the specific role they have chosen. This study allowed us to explore the extent to which the feeling of being constrained in fathers may have negative consequences on their coparental relationship, over and above objective characteristics such as the number of father’s or mother’s work hours. We hypothesized that when fathers feel constrained, coparenting will be less cohesive. To date, few—if any—studies have focused on the links between paternal motivations and coparenting.

## Materials and Methods

### Overview

The current study was part of a larger multisite and multinational study on parental burnout in different countries throughout the world conducted by an international consortium (International Investigation of Parental Burnout) led by the Catholic University of Louvain. Coparenting and motivation for role distribution were not surveyed in the general study; these variables were added to the Swiss part of the survey. As no specific instrument was available to assess motivation for role distribution, we created a questionnaire specifically designed for this study for exploratory purposes: Motivations for Role Distribution (MRD). Coparenting was assessed with the Coparenting Relationship Scale (CRS), a seven-dimension questionnaire that has been well validated in English and in French (original version: Feinberg et al., 2012; French version: Favez et al., 2021b). Participants were individuals (mothers as well as fathers), not couples. For the present study, we focused on fathers only.

### Sample

The sample was a convenience sample of 144 fathers. Descriptive data on the sociodemographic characteristics of the sample are presented in Table 1.

**TABLE 1:** Descriptive statistics for sociodemographic variables (*N* = 143).

Variable	*M*	*SD*	Min.	Max.
Age (years)	40.97	7.59	25	62
Number of study years	16.63	3.74	6	29
Number of children	1.86	0.86	1	6
Work hours (%)	85.44	18.21	30	100
Wife/partner work hours (%)	72.69	32.54	0	100

*min, minimum; max, maximum.*

Regarding income, 100% of fathers were engaged in a paid activity, and 90.2% declared that they lived as a dual-income parenting couple. All fathers declared that they lived in a middle-class to upper-middle-class neighborhood. Regarding family structure, 86.1% (124) of fathers declared that they lived in a biparental house with the mother of their children, 8.3% (12) declared that they lived in a stepfamily, 4.9% (7) declared that they lived as a single parent, and 0.7% (1) declared that they lived in a same-sex family.

### Procedure

Recruitment was conducted through announcements in parents’ associations, public hospitals, and pediatric offices. We invited parents to answer an online questionnaire for which a link was provided. The study was completely anonymous, as we requested no data identifying the participants (e.g., name, date of birth). The study was conducted before the start of the coronavirus pandemic.

The general study received the approval of the Ethical Committee of the Catholic University of Louvain in Belgium. The specific Swiss part of the study received the approval of the Ethical Committee of the State of Vaud.

### Instruments

#### Coparenting

The coparenting relationship scale (CRS; Feinberg et al., 2012; French version: Favez et al., 2021b) contains 35 items along seven dimensions of coparenting. Four of these dimensions are worded in the positive or cohesive direction: “agreement” (four items, alpha = 0.82 in this study), “closeness” (five items, alpha = 0.80), “support” (six items, alpha = 0.93), and “endorsement of partner’s parenting” (seven items, alpha = 0.90). Three dimensions are worded in the negative or non-cohesive direction: “exposure to conflict” (five items, alpha = 0.90), “undermining” (six items, alpha = 0.86), and “division of labor” (two items, alpha = 0.50). Each item is assessed on a 7-point scale ranging from 0 (*not true of us*) to 6 (*very true of us*), except for items in the exposure to conflict dimension, for which items are assessed on a 7-point scale ranging from 0 (*never*) to 6 (*very often—several times a day*). Scores are obtained for each dimension by computing the means of the related items. Given its low internal consistency, the division of labor dimension was not considered in the analyses.

#### Motivations for Role Distribution

Eight items answered the question, “How did you decide on role distribution?”: (1) I did not have a choice for economic reasons; (2) I did not have a choice for practical reasons (e.g., it was difficult to find childcare); (3) it was evident that this is how I wanted things to be; (4) I would have preferred something else, but social pressure made us adopt this kind of sharing; (5) in my domain, reducing work hours is difficult because of work constraints; (6) I felt that diminishing my work hours could give a negative image of me to my employer; (7) I felt that my close relatives would judge me negatively if I had reduced my work hours to take care of my child; and (8) I felt that my close relatives would judge me negatively if I had not reduced my work hours to take care of my child. The items were assessed on 5-point Likert scales, with the following anchor points: 1 (*completely false*), 2, 3 (*neither true nor false*), 4, and 5 (*absolutely true*).

#### Sociodemographic Data

We used an *ad hoc* questionnaire to collect sociodemographic data: age of the fathers (in years), number of children living at home, study level (number of years successfully achieved), neighborhood (lower, middle, upper-middle), work hours of the father, work hours of the wife/partner.

### Statistical Analyses

A preliminary check was done to assess possible differences in coparenting according to family structure and to select the families to be included in the study. A full set of descriptive statistics (including mean and standard deviation) was then computed for all variables of the study. We finally performed multivariate general linear models (GLMs) to study the links between the eight motivations for role distribution and the coparenting dimensions. Age, number of children, education level, work hours of the father, and work hours of the mother were entered as covariates. Given the 13 predictors to be included (eight motivations and five covariates), the necessary sample size to ensure 80% power with an effect size of 0.15 was *N* = 131 (Ellis, 2010). Effect size was set according to meta-analyses in the coparenting domain, which generally report small to moderate effect sizes. For example, the effect of coparenting problems on child internalizing and externalizing symptoms was shown to be between 0.11 and 0.24 (Teubert and Pinquart, 2010). To increase statistical power, we performed correlational analyses between the eight items about motivation for role distribution and the six coparenting dimensions in order to select the motivational variables to be included in the models. To be included, a motivational variable had to be correlated with at least one of the coparenting dimensions. The final model included eight predictors, allowing us to ensure 90% power. Regarding the coparenting dimensions, skewness was between –1.111 and 2.393, one dimension (undermining) being above the –/ + 2.0 threshold (Curran et al., 1996). We thus used a bootstrap on 1,000 samples to compute the parameter estimates (95% confidence interval), which is a robust method for non-normal data distribution (Efron and Tibshirani, 1993). Predictors were not multicollinear; average variance inflation factors were between 1.034 and 1.354, far below the maximum acceptable threshold of 5.0 (Chatterjee and Simonoff, 2013). No tolerance value was below 0.2. All statistical analyses were performed with IBM SPSS Statistics for Windows, version 26. Power was calculated by using G*Power software, version 3.1.9.6.

## Results

### Preliminary Check

Significant differences appeared on coparenting according to family structure for coparenting agreement, *F*(2, 140) = 6.48, *p* = 0.002; closeness, *F*(2, 140) = 14.38, *p* < 0.001; support, *F*(2, 140) = 7.34, *p* < 0.001; undermining, *F*(2, 140) = 6.23, *p* = 0.003; and endorsement of partner’s parenting, *F*(2, 140) = 12.56, *p* < 0.001. Contrasts showed that positive coparenting dimensions were significantly higher in biparental families than in single father families, and coparenting undermining was significantly higher in single father families than in biparental families and in stepfamilies. Coparental agreement, support, and endorsement of partner’s parenting were higher in stepfamilies than in single father families, coparenting closeness was higher in stepfamilies than in the two other types of families, and there was less exposure to conflict in stepfamilies than in biparental families. The same-sex family was not included in these comparisons, as there was only one family of this type. Given these results, further analyses were performed only in the group of biparental families, the family structure most represented in our sample, in order to avoid confound effects. One participant was further excluded for missing data. The final sample was thus 123 fathers living in a biparental family.

### Descriptive Analyses

Descriptive data for the motivations for role distribution and for coparenting (means and score ranges) are displayed in Table 2. The three motivations that fathers perceived as being most related to their decision on role distribution were economic reasons, practical reasons, and the decision being considered “evident” by fathers. The less influential motivations were the two related to close relatives. Regarding coparenting, the means for the cohesive dimensions (agreement, closeness, support, and endorsement of partner’s parenting) were situated on the higher end of the scales, whereas the means for the non-cohesive dimensions (exposure to conflict, undermining) were situated on the lower end of the scales, which is congruent with the nature of the sample (a non-clinical convenience sample).

**TABLE 2:** Descriptive statistics for motivations for role distribution and for coparenting (*N* = 123).

Variable	*M*	*SD*	Min.	Max.
*Motivations for role distribution*				
Economic reasons	3.15	1.28	1	5
Practical reasons	3.28	1.20	1	5
Evidence	3.22	1.23	1	5
Social pressure	2.04	1.25	1	5
Work constraint	2.67	1.49	1	5
Employer judgment	2.00	1.24	1	5
Close relatives judge reduction in work hours as negative	1.53	0.89	1	5
Close relatives judge no reduction in work hours as negative	1.85	1.19	1	5
*Coparenting Relationship Scale*				
Agreement	4.62	1.15	1.50	6.00
Closeness	3.85	1.18	1.00	6.00
Exposure to conflict	1.37	1.12	0.00	6.00
Support	4.07	1.51	0.00	6.00
Undermining	0.82	0.99	0.00	6.00
Endorsement of partner’s parenting	4.69	1.11	1.29	6.00

*min, minimum; max, maximum.*

Regarding the links between the control variables and coparenting, there was no significant correlation between the number of study years, the number of children, the father’s work hours, and any of the coparenting dimensions. On the other hand, the wife’s number of work hours was negatively correlated with endorsement of the partner’s parenting by the father (*r* = –0.185, *p* = 0.040). Age was also negatively correlated with coparenting closeness (*r* = –0.209, *p* = 0.020) and with coparenting support (*r* = –0.285, *p* < 0.001).

There were several links between control variables and motivations: item 1 (“I did not have a choice for economic reasons”) was positively correlated with the number of children (*r* = 0.208, *p* = 0.021); item 5 (“In my domain, reducing work hours is difficult because of work constraints”) was positively correlated with age (*r* = 0.230, *p* = 0.011), number of children (*r* = 0.179, *p* = 0.048), and number of work hours (*r* = 0.405, *p* < 0.001) and negatively correlated with the wife’s number of work hours (*r* = –0.274, *p* = 0.002). Item 7 (“I felt that my close relatives would judge me negatively if I had reduced my work hours to take care of my child”) was positively correlated with the number of study years (*r* = 0.193, *p* = 0.032) and number of children (*r* = 0.193, *p* = 0.032).

Correlations between motivations for role distribution and coparenting are provided in Table 3.

**TABLE 3:** Pearson two-tailed correlations between role distribution and coparenting (*N* = 123).

	1	2	3	4	5	6	7	8	9	10	11	12	13
1. MRD economic reasons	_												
2. MRD practical reasons	0.297**	_											
3. MRD evidence	–0.137	–0.197*	_										
4. MRD Social pressure	0.155	0.183*	–0.343***	_									
5. MRD Work constraints	–0.029	0.023	–0.105	0.073	_								
6. MRD Employer judgment	–0.073	0.121	–0.043	0.281**	0.434***	_							
7. MRD close relatives judge reduction in work hours as negative	–0.072	0.046	–0.115	0.179*	0.206*	0.522***	_						
8. MRD Close relatives judge no reduction in work hours as negative	0.064	0.019	–0.128	0.290**	0.036	0.206*	0.409***	_					
9. CRS agreement	–0.175	–0.012	0.251**	–0.288**	–0.020	0.049	0.098	–0.134	_				
10. CRS closeness	–0.153	–0.054	0.143	–0.071	–0.065	0.008	–0.021	–0.187*	0.559***	_			
11. CRS exposure conflict	0.046	–0.073	–0.094	0.196*	0.088	0.083	–0.002	0.180*	–0.584***	–0.430***	_		
12. CRS support	–0.144	–0.075	0.088	–0.189*	–0.127	–0.053	–0.066	–0.106	0.601***	0.660***	–0.551***	_	
13. CRS undermining	0.054	–0.095	–0.132	0.247**	0.096	0.086	–0.007	0.106	–0.666***	–0.424***	0.705***	–0.590***	_
14. CRS endorsement	–0.129	–0.024	0.181*	–0.179*	–0.033	0.026	0.046	–0.088	0.596***	0.561***	–0.418***	0.676***	–0.486***

*MRD, Motivations for Role Distribution; CRS, Coparenting Relationship Scale.*

*p < 0.05. **p < 0.01. ***p < 0.001.

MRD item 3 (“It was evident that this is how I wanted things to be”) was positively and significantly correlated with coparental agreement and endorsement of the partner’s parenting; item 4 (“I would have preferred something else, but social pressure made us adopt this kind of sharing”) was negatively correlated with the dimensions of coparental agreement, coparental support, and endorsement of the partner’s parenting, whereas it was positively correlated with exposure to conflict and coparental undermining; and item 8 (“I felt that my close relatives would judge me negatively if I had not reduced my work hours to take care of my child”) was negatively correlated with coparenting closeness and positively correlated with exposure to conflict. These correlations were all coherent: item 3 related to voluntary choice was positively correlated with a functional dimension of coparenting, whereas the two items related to a felt constraint or social pressure (items 4 and 8) were negatively correlated with functional dimensions of coparenting and positively correlated with dysfunctional dimensions of coparenting.

### Motivations as Predictors of Coparenting

Following the analysis of the correlations, items 3, 4, and 8 of the MRD were selected for GLM analyses. Results of the multivariate GLM performed on the six coparenting dimensions showed first, that age is the only predictor of all coparenting dimensions taken together, *F*(6, 109) = 2.759, *p* = 0.016. Parameter estimates (see Table 4), on the other hand, showed several links between the predictors and separate dimensions of coparenting. Age was a negative predictor of coparenting closeness, support, and endorsement of the partner’s parenting.

**TABLE 4:** Parameter estimates of the effects of control variables and role distribution on coparenting (*N* = 123).

	CRS agreement				CRS closeness				CRS exposure to conflict			
			95% CI				95% CI				95% CI	
Parameter	*B*	*SE*	*LL*, *UL*	*p*	*B*	*SE*	*LL*, *UL*	*p*	*B*	*SE*	*LL*, *UL*	*p*
Intercept	5.155	0.989	3.044, 6.995	**0.001**	5.409	1.034	3.456, 7.435	**0.001**	1.091	0.950	–0.846, 2.958	0.258
Work hours	0.004	0.007	–0.009, 0.017	0.581	0.003	0.008	–0.013, 0.018	0.671	–0.003	0.007	–0.016, 0.012	0.723
Wife/partner work hours	–0.004	0.004	–0.011, 0.003	0.260	–0.003	0.004	–0.010, 0.004	0.352	0.002	0.004	–0.007, 0.010	0.704
Age	–0.018	0.015	–0.047, 0.010	0.232	–0.038	0.016	–0.068, –0.007	**0.018**	0.000	0.014	–0.029, 0.027	0.994
Number of study years	0.007	0.028	–0.052, 0.062	0.814	–0.008	0.025	–0.061, 0.038	0.751	–0.007	0.032	–0.070, 0.056	0.837
Number of children	–0.038	0.131	–0.275, 0.245	0.744	–0.087	0.139	–0.408, 0.145	0.506	0.067	0.118	–0.162, 0.324	0.564
MRD Evidence	0.187	0.090	0.009, 0.373	**0.038**	0.153	0.091	–0.024, 0.329	0.098	–0.027	0.082	–0.199, 0.133	0.748
MRD Social pressure	–0.184	0.098	–0.372, 0.005	0.061	0.036	0.096	–0.140, 0.232	0.741	0.126	0.085	–0.041, 0.296	0.139
MRD Close relatives judge no reduction in work hours as negative	–0.040	0.105	–0.240, 0.174	0.697	–0.180	0.093	–0.351, 0.008	0.058	0.114	0.093	–0.071, 0.286	0.228

		**CRS support**				**CRS undermining**				**CRS endorsement**			
			
			**95% CI**				**95% CI**				**95% CI**	
**Parameter**	***B***	***SE***	***LL, UL***	***p***	***B***	***SE***	***LL, UL***	***p***	***B***	***SE***	***LL, UL***	***p***

Intercept	6.324	1.358	3.531, 8.884	0.001	0.260	0.819	–1.233, 2.117	0.741	5.751	0.932	3.969, 7.681	**0.001**
Work hours	0.007	0.011	–0.014, 0.029	0.556	0.000	0.006	–0.012, 0.011	0.944	0.006	0.008	–0.009, 0.022	0.489
Wife/partner work hours	0.000	0.005	–0.010, 0.011	0.979	–0.002	0.004	–0.010, 0.006	0.672	–0.008	0.004	–0.015, 0.000	0.051
Age	–0.062	0.018	–0.097, –0.025	**0.004**	0.021	0.011	–0.001, 0.042	0.067	–0.033	0.013	–0.058, –0.005	**0.025**
Number of study years	0.010	0.037	–0.062, 0.083	0.795	–0.010	0.029	–0.070, 0.044	0.747	0.009	0.026	–0.045, 0.064	0.723
Number of children	–0.081	0.161	–0.442, 0.210	0.593	–0.119	0.091	–0.298, 0.061	0.196	–0.080	0.128	–0.345, 0.168	0.500
MRD Evidence	0.063	0.119	–0.166, 0.300	0.601	–0.056	0.082	–0.236, 0.087	0.514	0.167	0.078	0.009, 0.322	**0.034**
MRD Social pressure	–0.177	0.132	–0.422, 0.101	0.187	0.169	0.074	0.016, 0.313	**0.030**	–0.085	0.090	–0.252, 0.096	0.347
MRD Close relatives judge no reduction in work hours as negative	–0.083	0.124	–0.327, 0.154	0.513	0.047	0.085	–0.118, 0.207	0.603	–0.020	0.075	–0.170, 0.124	0.762

*These parameters are bootstrap estimates (n = 1,000 samples).*

*MRD, Motivations for Role Distribution; CRS, Coparenting Relationship Scale; CI, confidence interval; LL, lower limit; UL, upper limit.*

*Boldface: p significant below the threshold of 0.05.*

Evidence was a positive predictor of coparenting agreement and of endorsement of the partner’s parenting; social pressure was a positive predictor of coparenting undermining. None of the other variables (work hours, wife/partner work hours, number of study years, number of children, work constraints, and expectations by close relatives) were predictors of any of the coparenting dimensions.

## Discussion

The aim of this study was to assess the extent to which motivations and expectations of fathers about role distribution were predictive of the coparenting relationship that they report to have with the mother. Our results show that having chosen a specific role as evident, that is, in line with the father’s will, is predictive of better agreement in coparenting and greater endorsement of the partner’s parenting. In contrast, having chosen a role because of social pressure (whether effective or perceived as such) is linked with more undermining behaviors, that is, the feeling of being more at ease with the children when the mother is not present and the report of more negative behaviors made by the mothers in the coparenting interactions.

Several variables related to objective stressors were also considered, such as the number of work hours, the number of wife/partner work hours, and the number of children. These variables have been shown to be linked with the burden felt in the imbalance in work-family duties (Allen et al., 2013) and may thus indirectly affect the coparental relationship, in particular through a spillover effect. However, none of these variables were linked with coparenting. It is particularly interesting to note that neither the work rate of the fathers nor the work rate of the mothers reported by the fathers is predictive of coparenting. In most studies, paternal engagement has indeed been assessed in terms of time distribution; several meta-analyses have shown that the number of work hours is positively related to work-family conflicts (Byron, 2005; Ng and Feldman, 2008), and so we could expect it to have consequences on coparenting as well, which was not the case. Our results are in line with those of studies showing that the perception of stress rather than the actual workload may generate work-family conflicts (Perry-Jenkins et al., 2000). Alternatively, our results may also indicate that the processes that explain work-family conflicts and within-family conflicts related to work (coparenting difficulties fall into the domain of within-family difficulties) are not completely equivalent. However, further studies are needed to test these possible differences.

Not only is the number of work hours unrelated to coparenting, but there is also no correlation between the number of work hours and any of the motivational items. This finding suggests that there is no linear association between the amount of time at work and the feeling of being constrained. One of the reasons for this absence of a link may be the variety in fathers’ expectations: not all fathers want to be the breadwinner of the family (to conform with the traditional role), but not all fathers want to be engaged in family work (as the zeitgeist could push them to do). Both a father dedicated to work and a father engaged in family life may feel constrained by social expectations; some fathers working full time may feel forced to do so, and others working part time and engaged in family duties may have the same feeling. This may reflect individual differences, as well as the ambivalence in contemporaneous social demands, according to which one should be at the same time a successful worker and an efficient caregiver—the negative consequences of which have been highlighted in mothers (Borelli et al., 2017). On the one hand, fathers may be willing to engage themselves more in family life, but they may have to face negative opinions from their social network (they may be presumed to be unable to get a paid job, or be unable to take care of children, or even be harmful to them; Rochlen et al., 2010). To overcome these criticisms, some fathers report rebuilding their masculine identity by incorporating feminine qualities (such as caregiving) in the definition of being masculine (caregiving is seen as an alternate way to provide resources to the family; Lee and Lee, 2018). In our own studies, we have found that when fathers endorse feminine traits (being affectionate), coparenting interactions are of higher quality (Favez and Frascarolo, 2020; Favez et al., 2021a). On the other hand, some fathers may still be more at ease with the traditional role of breadwinner, but in this case, they fail to meet the expectations of the mothers about paternal engagement (Fox et al., 2000). The variety of expectations in fathers may be a sign of social change toward roles that are less determined by biological sex and more related to an individual’s wishes and desires, or to personality factors such as masculine and feminine traits (Donnelly and Twenge, 2017). However, this process may be especially slow to take hold in Switzerland, a country in which traditional role distribution is still strongly predominant and structural support of families limited in comparison with that of other European countries (Bonoli, 2007; Levy and Widmer, 2013). As a consequence, it is difficult for mothers to depart from their role as primary housekeeper and caretaker, and for fathers to diminish their investment in work in order to be more available for family life.

The feeling of being constrained may be linked with a sense of inequity that will negatively affect the coparenting dynamic, as the father may have the feeling of doing a lot and not receiving his share (DeMaris and Mahoney, 2017). This explanation is speculative, however, as we did not assess the feeling of inequity in our study and therefore cannot verify the accuracy of this process. On the other hand, a feeling of evidence in the way roles were distributed is linked to coparental agreement and to the endorsement of the partner’s parenting. This positive link may be the mere consequence of a distribution that met the father’s will; however, it would be interesting to investigate the extent to which a general positive attitude may explain both the feeling of evidence (fathers are happy with what they have) and cohesion in the coparental relationship. This second option would be in line with the role-enhancement perspective according to which positive affect and state of mind is an antecedent of mutual enrichment between work and family domains (Michel et al., 2011a; Lapierre et al., 2018). Further studies would be needed to test this hypothesis, as well as to assess the links between the feeling of evidence and satisfaction with work.

Whereas the number of work hours is not related to coparenting, another sociodemographic variable is a strong predictor of the relation between the parents: the age of the fathers. The older the father is, the less he reports coparenting closeness with the mother, coparenting support, and endorsement of the mother’s parenting. The influence of age reminds us of the importance of considering the life cycle of families. In our study, we used the age of the father as a predictor, which is strongly positively correlated with the ages of the older child and of the younger child of the family (including all three variables would have inflated the results related to age). Coparenting does not have the same meaning and the same aim for the different developmental stages of the children; when the child is very young, support is all the more important at an instrumental level, for example. Although there is no specific theory on the life trajectory of coparenting, studies have shown that some positive dimensions related to cohesion are less active as the child grows (Favez et al., 2015): there is less and less promotion of family integrity, for example. This observation may be explained by the necessity for the family to be progressively more open in order to allow the child to develop relationships with family outsiders and not to feel stuck within the family, this being similar to the “enmeshed” configuration described in some problematic families (Minuchin, 1974). Interestingly, an effect of life cycle has also been found in work-family conflicts, which decrease as individuals age (Hill et al., 2014).

Our study has several limitations, the first of these related to the sample: The sample size is small, and we had to reduce it further because the family structures were linked to coparenting. We have thus focused on the most represented arrangement (86.1%), that is, a heterosexual biparental house with the biological children of the couples. Moreover, it was a convenience sample, and so it is not representative of the general population. The participants were individuals, not couples, the reason being that the main study was designed as an anonymous survey that targeted any and all parents interested in participating. It is thus possible that both partners in a couple completed the survey, but we had no means of knowing whether this was the case. It will be necessary to collect such data about motivations in role distribution in both partners in order to assess possible incongruencies or contradictions between their reports. This study was in fact an ancillary study, congruent with the aim of the main study, but not its main aim. For this reason, we were not able to enroll couples. Second, there are limitations related to the instruments: The questionnaire that we used to assess motivation for role distribution was created *ad hoc* for this study, as no questionnaire on this topic was available in the literature; more data are thus necessary to test its validity. Moreover, the division of labor dimension of the CRS, which is closely related to role distribution, was not included in the study due to its low internal consistency. Future studies should include questionnaires specifically dedicated to division of labor, such as the “Who does what” questionnaire (Cowan and Cowan, 1990). Finally, it would have been interesting to include an assessment of the couple relationship at a romantic (or marital) level, as the romantic and coparental facets of the couple relationship are deeply intertwined (see, for example, Fagan and Lee, 2014); dissatisfaction with the marital relationship may also explain a less cohesive coparental relationship.

Despite these limitations, and considering that the aim of this study was mainly exploratory, it has nevertheless shown that fathers may have different motivations and expectations about role distribution, and when their expectations are not met, this may have an impact on the coparental relationship. Both parents’ expectations and needs are thus to be considered, as this will strengthen parental alliance and coparental cohesion, which will in turn also be beneficial to mothers. In Europe, social policies vary greatly between countries, for example, regarding access to and duration of paternity leave; fathers should benefit from the same support and information as mothers (the vast majority of resources available for new parents being focused on the child and mothers only; Lee et al., 2020) and their expectations should be heard, as this will contribute to the well-being of the whole family.

## Data Availability Statement

The raw data supporting the conclusions of this article will be made available by the authors, without undue reservation.

## Ethics Statement

The studies involving human participants were reviewed and approved by the Ethical Committee of the State of Vaud. Written informed consent for participation was not required for this study in accordance with the national legislation and the institutional requirements.

## Author Contributions

NF designed and co-conducted the study, wrote the main parts of the manuscript, and made the statistical analyses along with HT. AM participated in designing the study, collected the data, and built the database. MB designed and co-conducted the study. HT made the statistical analyses and co-wrote parts of the manuscript. All authors contributed to the article and approved the submitted version.

## Conflict of Interest

The authors declare that the research was conducted in the absence of any commercial or financial relationships that could be construed as a potential conflict of interest.

## Publisher’s Note

All claims expressed in this article are solely those of the authors and do not necessarily represent those of their affiliated organizations, or those of the publisher, the editors and the reviewers. Any product that may be evaluated in this article, or claim that may be made by its manufacturer, is not guaranteed or endorsed by the publisher.

## References

[B1] Allen T. D. Herst D. E. L. Bruck C. S. Sutton M. (2000). Consequences associated with work-to-family conflict: a review and agenda for future research. *J. Occup. Health Psychol.* 5 278–308. 10.1037/1076-8998.5.2.278 10784291

[B2] Allen T. D. Johnson R. C. Kiburz K. M. Shockley K. M. (2013). Work–family conflict and flexible work arrangements: deconstructing flexibility. *Pers. Psychol.* 66 345–376. 10.1111/peps.12012

[B3] Amstad F. T. Meier L. L. Fasel U. Elfering A. Semmer N. K. (2011). A meta-analysis of work–family conflict and various outcomes with a special emphasis on cross-domain versus matching-domain relations. *J. Occup. Health Psychol.* 16 151–169. 10.1037/a0022170 21280939

[B4] Barnett R. C. Hyde J. S. (2001). Women, men, work, and family: an expansionist theory. *Am. Psychol.* 56 781–796. 10.1037/0003-066X.56.10.781 11675985

[B5] Bianchi S. M. Milkie M. A. (2010). Work and family research in the first decade of the 21st century. *J. Marriage Fam.* 72 705–725. 10.1111/j.1741-3737.2010.00726.x

[B6] Bodenmann G. Ledermann T. Bradbury T. N. (2007). Stress, sex, and satisfaction in marriage. *Pers. Relatsh.* 14 551–569. 10.1111/j.1475-6811.2007.00171.x

[B7] Bonoli G. (2007). Time matters: postindustrialization, new social risks, and welfare state adaptation in advanced industrial democracies. *Comp. Political Stud.* 40 495–520. 10.1177/0010414005285755

[B8] Borelli J. L. Nelson S. K. River L. M. Birken S. A. Moss-Racusin C. (2017). Gender differences in work-family guilt in parents of young children. *Sex Roles* 76 356–368. 10.1007/s11199-016-0579-0

[B9] Byron K. (2005). A meta-analytic review of work–family conflict and its antecedents. *J. Vocat. Behav.* 67 169–198. 10.1016/j.jvb.2004.08.009

[B10] Carlson D. L. Petts R. J. Pepin J. R. (2021). Flexplace work and partnered fathers’ time in housework and childcare. *Men Masc.* 24 547–570. 10.1177/1097184x211014929

[B11] Chatterjee S. Simonoff J. S. (2013). *Handbook of Regression Analysis.* Hoboken, NJ: Wiley.

[B12] Cowan C. P. Cowan P. A. (1990). “Who does what?” *Handbook of Family Measurement Techniques*eds Touliatos J. Perlmutter B. Straus M. (Newbury Park, CA: Sage), 278–279.

[B13] Cowan C. P. Cowan P. A. (1992). *When Partners Become Parents: The Big Life Change for Couples.* Mahwah, NJ: Lawrence Erlbaum.

[B14] Craig L. (2006). Does father care mean fathers share? A comparison of how mothers and fathers in intact families spend time with children. *Gend. Soc.* 20 259–281. 10.1177/0891243205285212

[B15] Curran P. J. West S. G. Finch J. F. (1996). The robustness of test statistics to nonnormality and specification error in confirmatory factor analysis. *Psychol. Methods* 1 16–29. 10.1037/1082-989X.1.1.16

[B16] DeMaris A. Mahoney A. (2017). Equity dynamics in the perceived fairness of infant care. *J. Marriage Fam.* 79 261–276. 10.1111/jomf.12331 28082750 PMC5221818

[B17] Demerouti E. Bakker A. B. Schaufeli W. B. (2005). Spillover and crossover of exhaustion and life satisfaction among dual-earner parents. *J. Vocat. Behav.* 67 266–289. 10.1016/j.jvb.2004.07.001

[B18] DeRose L. F. Goldscheider F. Brito J. R. Salazar-Arango A. Corcuera P. Corcuera P. J. (2019). Are children barriers to the gender revolution? International comparisons. *Eur. J. Popul.* 35 987–1021. 10.1007/s10680-018-09515-8 31832033 PMC6882994

[B19] Dew J. Wilcox W. B. (2011). If momma ain’t happy: explaining declines in marital satisfaction among new mothers. *J. Marriage Fam.* 73 1–12. 10.1111/j.1741-3737.2010.00782.x

[B20] Donnelly K. Twenge J. M. (2017). Masculine and feminine traits on the Bem Sex-Role Inventory, 1993–2012: a cross-temporal meta-analysis. *Sex Roles* 76 556–565. 10.1007/s11199-016-0625-y

[B21] Efron B. Tibshirani R. (1993). *An Introduction to the Bootstrap.* New York, NY: Chapman & Hall.

[B22] Ellis P. D. (2010). *The Essential Guide to Effect Sizes: Statistical Power, Meta-analysis, and the Interpretation of Research Results.* Cambridge, MA: University Press.

[B23] Fagan J. Lee Y. (2014). Longitudinal associations among fathers’ perception of coparenting, partner relationship quality, and paternal stress during early childhood. *Fam. Process* 53 80–96. 10.1111/famp.12055 24236848

[B24] Favez N. Frascarolo F. (2020). Gender-role orientation in parents: a factor contributing to prenatal coparental interactions in primiparous families. *Early Child Dev. Care* 190 2181–2191. 10.1080/03004430.2018.1564915

[B25] Favez N. Tissot H. Golay P. Max A. Feinberg M. E. Bader M. (2021b). French adaptation of the coparenting relationship scale: a scale for the assessment of the interparental relationship. *Eur. J. Psychol. Assess* 37 1–6. 10.1027/1015-5759/a000633

[B26] Favez N. Tissot H. Frascarolo F. (2021a). Shared parental care in the first 18 months as a context for sensitivity and coparenting. *J. Fam. Stud.* 27 215–230. 10.1080/13229400.2018.1527711

[B27] Favez N. Widmer E. Doan M.-T. Tissot H. (2015). Coparenting in stepfamilies: maternal promotion of family cohesiveness with partner and with father. *J. Child Fam. Stud.* 24 3268–3278. 10.1007/s10826-015-0130-x

[B28] Federal Statistical Office (2021). *Women did 50% More Domestic And Family Work Than Men In 2020-But Men Catching Up.* Available online at: https://www.bfs.admin.ch/bfs/fr/home/actualites/quoi-de-neuf.assetdetail.17124479.html (accessed October 15, 2021).

[B29] Feinberg M. E. (2003). The internal structure and ecological context of coparenting: a framework for research and intervention. *Parenting* 3 95–131. 10.1207/s15327922par0302_01 21980259 PMC3185375

[B30] Feinberg M. E. Brown L. D. Kan M. L. (2012). A multi-domain self-report measure of coparenting. *Parenting* 12 1–21. 10.1080/15295192.2012.638870 23166477 PMC3499623

[B31] Fox G. L. Bruce C. Combs-Orme T. (2000). Parenting expectations and concerns of fathers and mothers of newborn infants. *Fam. Relat.* 49 123–131.

[B32] Fraenkel P. Capstick C. (2012). “Contemporary two-parent families: navigating work and family challenges,” in *Normal Family Processes: Growing Diversity and Complexity*, 4th Edn, ed. Walsh F. (New York, NY: The Guilford Press), 78–101.

[B33] Frejka T. Goldscheider F. Lappegård T. (2018). The two-part gender revolution, women’s second shift and changing cohort fertility. *Comp. Popul. Stud.* 43 99–130.

[B34] Goldberg A. E. Perry-Jenkins M. (2004). Division of labor and working-class women’s well-being across the transition to parenthood. *J. Fam. Psychol.* 18 225–236. 10.1037/0893-3200.18.1.225 14992623 PMC2834188

[B35] Goldscheider F. Bernhardt E. Lappegård T. (2015). The gender revolution: a framework for understanding changing family and demographic behavior. *Popul. Dev. Rev.* 41 207–239. 10.1111/j.1728-4457.2015.00045.x

[B36] Goode W. J. (1960). A theory of role strain. *Am. Sociol. Rev.* 25 483–496.

[B37] Goodman J. H. (2005). Becoming an involved father of an infant. *J. Obst. Gynecol. Neonatal Nurs.* 34 190–200. 10.1177/0884217505274581 15781596

[B38] Gottfried A. E. Gottfried A. W. Bathurst K. (2002). “Maternal and dual-earner employment status and parenting,” in *Handbook of Parenting: Biology and Ecology of Parenting*, 2nd Edn, Vol. 2 ed. Bornstein M. (Hillsdale, NJ: Lawrence Erlbaum Associates Publishers), 207–229.

[B39] Greenhaus J. H. Beutell N. (1985). Sources of conflict between work and family roles. *Acad. Manage. Rev.* 10 76–88.

[B40] Greenhaus J. H. Powell G. N. (2006). When work and family are allies: a theory of work-family enrichment. *Acad. Manage. Rev.* 31 72–92.

[B41] Hill E. J. Holmes E. K. (2019). “Families and workplaces,” in *APA Handbook of Contemporary Family Psychology. Applications and Broad Impacts of Family Psychology*, Vol. 2 ed. Fiese B. (Washington, DC: American Psychological Association), 379–395.

[B42] Hill E. J. Erickson J. J. Fellows K. J. Martinengo G. Allen S. M. (2014). Work and family over the life course: do older workers differ? *J. Fam. Econ. Issues* 35 1–13. 10.1007/s10834-012-9346-8

[B43] Hogan V. Hogan M. Hodgins M. Kinman G. Bunting B. (2014). An examination of gender differences in the impact of individual and organisational factors on work hours, work-life conflict and psychological strain in academics. *Ir. J. Psychol.* 35 133–150. 10.1080/03033910.2015.1011193

[B44] Jacobs J. N. Kelley M. L. (2006). Predictors of paternal involvement in childcare in dual-earner families with young children. *Fathering* 4 23–47. 10.3149/fth.0401.23

[B45] Jobin C. (1995). *Entre Les Activités Professionnelle Et Domestique: La Discrimination Sexuelle [Between Professional And Home Occupation: The Sexual Discrimination].* Lausanne: Editions d’en bas.

[B46] Lamb M. E. Lewis C. (2010). “The development of significance of father-child relationships in two-parent families,” in *The Role of the Father in Child Development*, ed. Lamb M. E. (Hoboken, NJ: Wiley), 94–153.

[B47] Lamb M. E. Tamis-LeMonda C. S. (2004). “The role of the father: an introduction,” in *The Role of the Father in Child Development*, ed. Lamb M. E. (Hoboken, NJ: John Wiley & Sons), 1–31.

[B48] Lapierre L. M. Li Y. Kwan H. K. Greenhaus J. H. DiRenzo M. S. Shao P. (2018). A meta-analysis of the antecedents of work–family enrichment. *J. Organ. Behav.* 39 385–401. 10.1002/job.2234

[B49] Lee J. Y. Lee S. J. (2018). Caring is masculine: stay-at-home fathers and masculine identity. *Psychol. Men Masc.* 19 47–58. 10.1037/men0000079

[B50] Lee S. J. Lee J. Y. Chang O. D. (2020). “The characteristics and lived experiences of modern stay-at-home fathers,” in *Handbook of Fathers and Child Development: Prenatal to Preschool*, eds Fitzgerald H. E. von Klitzing K. Cabrera N. J. Scarano de Mendonça J. Skjøthaug T. (Cham: Springer International Publishing), 537–549. 10.1007/978-3-030-51027-5_32

[B51] Levy R. Widmer E. D. (eds) (2013). *Gendered Life Courses Between Standardization and Individualization: A European Approach Applied to Switzerland*, Vol. 18. Zurich: LIT Verlag.

[B52] Maurer T. Pleck J. Rane T. (2001). Parental identity and reflected-appraisals: measurement and gender dynamics. *J. Marriage Fam.* 63 309321. 10.1111/j.1741-3737.2001.00309.x

[B53] McGill B. S. (2014). Navigating new norms of involved fatherhood: employment, fathering attitudes, and father involvement. *J. Fam. Issues* 35 1089–1106. 10.1177/0192513X14522247

[B54] McHale J. P. (2007). *Charting the Bumpy Road of Coparenthood: Understanding the Challenges of Family Life.* Washington, DC: Zero to Three.

[B55] McHale J. P. Lindahl K. (eds) (2011). *Coparenting: A Conceptual and Clinical Examination of Family Systems.* Washington, DC: American Psychological Association.

[B56] Michel J. S. Kotrba L. M. Mitchelson J. K. Clark M. A. Baltes B. B. (2011b). Antecedents of work–family conflict: a meta-analytic review. *J. Organ. Behav.* 32 689–725. 10.1002/job.695

[B57] Michel J. S. Clark M. A. Jaramillo D. (2011a). The role of the Five Factor Model of personality in the perceptions of negative and positive forms of work–nonwork spillover: a meta-analytic review. *J. Vocat. Behav.* 79 191–203. 10.1016/j.jvb.2010.12.010

[B58] Milkie M. A. Bianchi S. M. Mattingly M. J. Robinson J. P. (2002). Gendered division of childrearing: ideals, realities, and the relationship to parental well-being. *Sex Roles* 47 21–38. 10.1023/A:1020627602889

[B59] Minuchin S. (1974). *Families and Family Therapy.* Cambridge, MA: Harvard University Press.

[B60] Moller K. Hwang P. C. Wickberg B. (2008). Couple relationship and transition to parenthood: does workload at home matter? *J. Reprod. Infant Psychol.* 26 57–68. 10.1080/02646830701355782

[B61] National Institute of Child Health and Human Development (NICHD) Early Child Care Research Network (2000). Factors associated with fathers’ caregiving activities and sensitivity with young children. *J. Fam. Psychol* 14 200–219. 10.1037/0893-3200.14.2.200 10870290

[B62] Ng T. W. H. Feldman D. C. (2008). Long work hours: a social identity perspective on meta-analysis data. *J. Organ. Behav.* 29 853–880. 10.1002/job.536

[B63] Perälä-Littunen S. (2007). Gender equality or primacy of the mother? Ambivalent descriptions of good parents. *J. Marriage Fam.* 69 341–351. 10.1111/j.1741-3737.2007.00369.x

[B64] Perry-Jenkins M. Repetti R. L. Crouter A. C. (2000). Work and family in the 1990s. *J. Marriage Fam.* 62 981–998. 10.1111/j.1741-3737.2000.00981.x

[B65] Raley S. Bianchi S. M. Wang W. (2012). When do fathers care? Mothers’ economic contribution and fathers’ involvement in child care. *Am. J. Sociol.* 117 1422–1459. 10.1086/663354 26379287 PMC4568757

[B66] Rochlen A. B. McKelley R. A. Whittaker T. A. (2010). Stay-at-home fathers’ reasons for entering the role and stigma experiences: a preliminary report. *Psychol. Men Masc.* 11 279–285. 10.1037/a0017774

[B67] Steinhour H. (2018). I am not “Mr. Mom”: a qualitative analysis of at-home father’s struggle for legitimacy. *J. Gend. Stud.* 27 388–400. 10.1080/09589236.2016.1220290

[B68] Teubert D. Pinquart M. (2010). The association between coparenting and child adjustment: a meta-analysis. *Parenting* 10 286–307. 10.1080/15295192.2010.492040

[B69] Van Egeren L. A. Hawkins D. P. (2004). Coming to terms with coparenting: implications of definition and measurement. *J. Adult Dev.* 11 165–178. 10.1023/B:JADE.0000035625.74672.0b

[B70] Yeung W. J. Sandberg J. F. Davis-Kean P. E. Hofferth S. L. (2001). Children’s time with fathers in intact families. *J. Marriage Fam.* 63 136–154. 10.1111/j.1741-3737.2001.00136.x

